# Prevalence of non-HLA antibodies and their association with FIB-4 index-based fibrosis risk in pediatric liver transplant recipients with long-term graft survival (>10 years)

**DOI:** 10.3389/fimmu.2026.1813802

**Published:** 2026-06-15

**Authors:** Ricardo Cuesta-Martín de la Cámara, Andrea Rodrigo-Castro, Pilar Nozal, Carmen Cámara, Itsaso Losantos-García, Esteban Frauca-Remacha, Loreto Hierro-Llanillo, Gema Muñoz-Bartolo, María Dolores Lledín-Barbacho, Esther Mancebo, María José Castro-Panete, Estela Paz-Artal, Eduardo López-Granados, Elena Sánchez-Zapardiel

**Affiliations:** 1 Clinical Immunology Department, University Hospital La Paz, Madrid, Spain; 2 Lymphocyte Pathophysiology in Immunodeficiencies Group, La Paz Institute for Health Research (IdiPAZ), Madrid, Spain; 3 Medicine and Surgery Department, PhD School, Autonomous University of Madrid, Madrid, Spain; 4 Diagnosis and Treatment of Pathologies Associated with Alterations of the Complement System Group, La Paz Biomedical Research Institute (IdiPAZ), La Paz University Hospital, Madrid, Spain; 5 Rare Diseases Networking Biomedical Research Centre (CIBERER U754), Madrid, Spain; 6 Biostatistics Platform, La Paz Institute for Health Research (IdiPAZ), Madrid, Spain; 7 Pediatric Hepatology Department, University Hospital La Paz, Madrid, Spain; 8 European Reference Network (ERN) RARE LIVER, Madrid, Spain; 9 European Reference Network (ERN) TransplantChild, Madrid, Spain; 10 Immunology Department, University Hospital 12 de Octubre, Madrid, Spain; 11 12 de Octubre Hospital Health Research Institute (imas12), Madrid, Spain; 12 Department of Immunology, Ophthalmology and ENT, Complutense University School of Medicine, Madrid, Spain; 13 Biomedical Research Networking Center for Infectious Diseases (CIBERINFEC), Madrid, Spain; 14 Rare Diseases Networking Biomedical Research Centre (CIBERER U767), Madrid, Spain

**Keywords:** antibodies, graft rejection, HLA antigens, liver fibrosis risk, liver transplantation

## Abstract

**Background:**

While donor-specific antibodies against human leukocyte antigens (HLA-DSA) are recognized contributors to allograft injury, non-HLA antibodies are increasingly implicated in solid-organ transplant rejection. However, evidence in pediatric liver transplantation remains limited. We aimed to identify anti-HLA and non-HLA antibodies associated with liver fibrosis risk in long-term pediatric liver transplant recipients.

**Methods:**

This cross-sectional study included 75 pediatric liver recipients transplanted between 2006-2014 (median 13.8 years), 15 pediatric patients on liver-transplant waiting list and 22 healthy adult controls (HC). Serum samples were tested for non-HLA, anti-HLA class-I, class-II and anti-MHC class-I chain-related protein A (MICA) antibodies by multiplex microsphere-based flow cytometry (Luminex Technology). Liver fibrosis risk was assessed using the Fibrosis-4 (FIB-4) index. Univariate and multivariate logistic regression analyses evaluated associations between antibody positivity and FIB-4 index elevation.

**Results:**

Liver-transplant patients showed significantly higher frequencies of eight non-HLA specificities compared to HC: C-X-C motif chemokine ligand 9 (CXCL9), CXCL10, CXCL11, glyceraldehyde-3-phosphate dehydrogenase (GAPDH), glial cell line-derived neurotrophic factor (GDNF), leucine-rich repeat transmembrane protein 2 (FLRT2), interferon gamma (IFNG) and vimentin (VM). Anti-HLA class-II antibody prevalence was significantly higher post-transplantation (44% vs. 0%, p=0.002). In multivariate analysis, anti-GDNF antibody positivity was independently associated with increased FIB-4 values (odds ratio [OR] 5.61, p=0.04), while female sex showed a protective effect (OR 0.23, p=0.02).

**Conclusions:**

Eight non-HLA antibodies are prevalent in pediatric liver-transplant recipients and waitlisted candidates. Anti-GDNF antibodies were independently associated with higher FIB-4 values in long-term pediatric liver transplant recipients, suggesting a potential biomarker role in liver injury that requires validation in larger longitudinal cohorts.

## 1 Introduction

The deleterious impact of donor-specific antibodies directed against human leukocyte antigens (HLA-DSA) is well recognized in the context of solid organ transplantation (1–7). In kidney transplantation (KTx) this phenomenon is widely acknowledged, as preformed HLA-DSA can precipitate hyperacute rejection, whereas *de novo* HLA-DSA are primarily associated with acute and chronic antibody-mediated rejection (1). In contrast, the tolerogenic properties of the liver may have led to the assumption that this organ is relatively protected from the deleterious effects of alloimmunity (8). In fact, only a few cases of hyperacute rejection have been reported (9, 10).

However, accumulating evidence indicates that HLA-DSA may affect liver transplant (LTx) outcomes. In a cohort of 896 adult LTx recipients, Castillo-Rama et al. (11) demonstrated that preformed anti-HLA class-II antibodies were significantly linked to reduced survival at both 1 and 5 years post-transplantation. A few years later, O’Leary et al. (12) demonstrated in a retrospective study of 1,270 adult LTx recipients that preformed HLA-DSA class-II were associated with an increased risk of early graft rejection. Complementing these observations, Kaneku et al. (13) retrospectively showed in 749 adult LTx recipients that *de novo* HLA-DSA one year post-transplantation was linked to poorer graft and patient survival.

Long-term studies have further expanded our understanding of the association of alloantibodies on chronic LTx outcomes. In a seven-year prospective study of 123 adult LTx recipients, Ciszek et al. (14) found that the presence of either anti-HLA or anti-MHC class-I chain-related protein A (MICA) antibodies correlated with late graft rejection. Furthermore, emerging evidence has linked HLA-DSA to the development of post-transplant liver fibrosis. De Bello et al. (15) retrospectively analyzed 267 adult LTx recipients demonstrating that the Metavir fibrosis score was significantly higher in patients with HLA-DSA. Additionally, a prospective study involving 133 liver biopsies from adult LTx recipients revealed that HLA-DSA levels increase over time and are linked to liver fibrosis (16).

While HLA-DSA remain central to graft rejection, emerging evidence suggest a link between non-HLA antibodies and graft injury (17). These can be classified as alloantibodies against polymorphic molecules, like MICA, or autoantibodies targeting self-antigens, such as collagen. Additionally, classical autoantibodies associated to autoimmune hepatitis (AIH) – such as anti-nuclear antigen (ANA), anti-liver-kidney microsomal (LKM), anti-smooth muscle (ASMA) and anti-mitochondrial (AMA) antibodies – have also been associated with liver injury and rejection, but whether they contribute to pathogenesis or reflect underlying damage is still debated (18). Although some LTx recipients underwent transplantation for AIH, suggesting possible recurrence, *de novo* AIH have also been reported and are similarly associated with graft injury (19).

Current knowledge about non-HLA specificities comes from studies in non-liver solid organ transplantation. In a study of 12 adult KTx recipients, elevated levels of antibodies against glutathione S-transferase theta 1 (GSTT1), C-X-C motif chemokine ligands 10 and 11 (CXCL10, CXCL11) and heterogeneous nuclear ribonucleoprotein K (HNRNPK) were associated with early rejection, compared to 18 stable recipients (20). In lung transplantation, a retrospective analysis of 111 recipients demonstrated associations between both preformed and *de novo* non-HLA antibodies and chronic lung dysfunction (21). Other studies involving similar cohorts reported that the cumulative number of positive non-HLA antibodies correlated more strongly with graft injury (22, 23). In heart transplantation, elevated levels of antibodies against vimentin (VM), tubulin, lamin-A/C (LMNA) and apolipoprotein L2 showed an association with antibody-mediated rejection in 64 recipients (24). The association of anti-VM antibodies with rejection was confirmed in a separate cohort of 13 heart transplant recipients (25).

Evidence supporting the role of non-HLA antibodies in LTx is emerging, particularly in the context of re-transplantation and long-term outcomes. Illustrating this, Xu et al. (26, 27) retrospectively analyzed levels of autoantibodies to the angiotensin II type 1 receptor (AT1R) and to the C-terminal laminin-like globular domain of perlecan (LG3) in cohorts of 94 and 93 adult patients who had received a second LTx. The presence of either AT1R or LG3 antibodies was associated with higher risk of graft loss, and notably, their combination with other antibodies had a synergistic deleterious effect. In a cohort of 1,269 adult LTx recipients, O’Leary et al. (28) showed that while preformed non-HLA antibodies had no impact on outcomes, isolated *de novo* anti-AT1R and anti-endothelin 1 type A receptor (ETAR) antibodies were associated with increased graft rejection and fibrosis progression.

Although most studies focused on adult LTx recipients, evidence from the limited pediatric cohorts available suggests similar patterns. A cross-sectional study with 100 pediatric LTx recipients reported that C3d positive HLA-DSA was associated with higher graft-loss rates (29). Another cross-sectional study including 81 pediatric LTx recipients concluded that anti-HLA-DRB1 and AT1R antibodies were significantly increased in patients with advanced fibrosis (30). Lastly, Varma et al. (18) prospectively analyzed 89 pediatric LTx recipients and found that the presence of either ANA, ASMA, LKM or HLA-DSA class-II were significantly associated with graft inflammation.

Despite current findings, evidence on the role of anti-HLA and non-HLA antibodies in pediatric LTx recipients remains scarce. Long-term assessment of non-HLA specificities may facilitate early detection of hepatic fibrosis or chronic rejection. This cross-sectional study investigates anti-HLA and non-HLA antibodies potentially associated with liver fibrosis risk in pediatric LTx recipients transplanted over 10 years ago. Our findings should be considered exploratory and hypothesis generating, aimed at identifying potential associations between non-HLA antibodies and the risk of fibrosis in this specific population.

## 2 Methods

### 2.1 Patients and study design

Our cross-sectional study included 75 children (Post-Tx cohort) from University Hospital La Paz, who underwent LTx between 2006-2014. Serum samples were collected at least 10 years post-transplantation (median 13.8 years, interquartile range (IQR) [11.9-15.2]). Epidemiological and clinical variables including age, sex, diagnosis and type of transplantation were collected. Donor age was not available in our database and was therefore not included in the analysis.

Additionally, two more cohorts were included: 15 children in LTx waiting list (Pre-Tx cohort) and 22 adult healthy controls (HC). The HC group consisted of 22 anonymous blood donors from our institution’s blood bank (80% male and 20% female), with a median age of 59 years (IQR 45-70). Although liver biochemistry was not specifically available for this cohort, all participants were active donors meeting standard health requirements for blood donation, including the absence of known chronic infections or systemic diseases. Sex and age data were unavailable for 12 HC participants.

Transplant indication was categorized according to Díaz Fernandez et al. (31). All patients gave informed consent, approved by the ethics committee of our institution (reference PI-4000).

### 2.2 Evaluation of liver function and fibrosis risk

Liver function parameters were measured in both Pre-Tx and Post-Tx cohorts (Siemens). Liver fibrosis risk was evaluated using the Fibrosis-4 (FIB-4) index, a non-invasive scoring system that estimates the risk of liver fibrosis based on patient’s age, aspartate and alanine aminotransferase levels, and platelet count (32). Following the findings from Habash et al. (33), cut-off points of 0.20 and 3.03 were applied to rule out or rule in advanced fibrosis, respectively. Based on those thresholds, liver fibrosis risk was classified as unlikely (<0.20), indeterminate (0.20-3.03) or likely (≥3.03). Given that no Post-Tx patients reached the threshold of 3.03, we used ≥0.20 as a cut-off to distinguish patients in whom fibrosis was unlikely from those in whom it could not be ruled out. In this specific context, a FIB-4 value ≥0.20 does not indicate advanced fibrosis, but rather that fibrosis cannot be excluded according to established pediatric data.

### 2.3 Detection of autoantibodies and immunoglobulins

Patients were monitored for autoantibodies by indirect immunofluorescence (IIF). ANA were detected on HEp-2 cells (Euroimmun), while AMA, LKM, ASMA, anti-parietal cell (ACPA) and anti-GSTT1 antibodies were screened using triple rodent tissue (Euroimmun). Positivity was defined as titers above 1:160 dilution. Serum immunoglobulin IgG, IgA and IgM levels were quantified by immunoassay (Siemens).

### 2.4 Detection of non-HLA antibodies

Serum samples from Pre-Tx and Post-Tx cohorts and plasma samples from HC were tested by multiplex microsphere-based flow cytometry (Luminex Technology, LABScreen™ Autoantibody Group 1, One Lambda), following manufacturer’s instructions, assessing 32 non-HLA specificities.

Positivity was defined based on the median fluorescence intensity (MFI) corresponding to the 95^th^ percentile of a reference cohort of non-transplanted individuals, as provided by the manufacturer. Given specificity-dependent MFI cut-off values depending on the antibody, a normalized MFI (nMFI) was calculated by dividing the observed MFI by its corresponding cut-off, with nMFI >1 indicating positivity.

### 2.5 Detection of anti-HLA and anti-MICA antibodies

Serum samples from patient cohorts were tested for anti-HLA class-I, anti-HLA class-II and anti-MICA antibodies by multiplex microsphere-based flow cytometry (Luminex Technology, LABScreen™ Mix, One Lambda), following manufacturer’s instructions. Samples with standard fluorescence intensity >15,000 were considered positive. Because donor HLA typing was not integrated into this analysis, HLA-DSA could not be identified, and reactivity is reported as anti-HLA class I or II antibody positivity.

### 2.6 Statistical analysis

Quantitative variables are presented as median with IQR, and qualitative variables as counts and proportions (%). Group comparisons used Mann-Whitney U or Student’s t-test, based on data normality assessed by Shapiro-Wilk test. Comparisons among three groups used Kruskal-Wallis test or one-way ANOVA. Associations between categorical variables were analyzed using Chi-square test or Fisher’s exact test. Correlations between continuous variables were assessed using Pearson’s correlation coefficient. Variables with a p-value <0.10 in the univariate logistic regression model were subsequently included in the multivariate model. To ensure model stability and prevent overfitting, the final multivariate model was constructed using 55 events (patients with FIB-4 value ≥0.20) and 2 variables (female sex and anti-GDNF positivity), maintaining an events-per-variable ratio greater than 10. Analyses across the 32 non-HLA specificities were exploratory and no formal adjustment for multiple testing was applied, which may increase the risk of type I error. A p-value <0.05 was considered statistically significant. All analyses were conducted with RStudio (version 4.3.3, R Core Team, 2024).

## 3 Results

### 3.1 Cohort description and clinical parameters

Demographic and clinical data of Post-Tx and Pre-Tx cohorts are summarized in **Table 1**. Seventy-five patients were included in the Post-Tx cohort, with a sex distribution of 45% males and 55% females, and a median age of 15 years (IQR 14-16). Ninety-one percent of the Post-Tx cohort were recipients of primary grafts. Graft rejection was previously diagnosed in 5% of patients. The most common immunosuppressive treatment was tacrolimus plus corticosteroids (52%).

**Table 1:** Epidemiologic and clinical features in two independent cohorts of pediatric liver transplant candidates (Pre-Tx) and long-term liver transplant recipients (Post-Tx).

Characteristics	Pre-Tx (n=15)	Post-Tx (n=75)	P-value
**Sex, n (%)**			0.78
Male	6 (40)	34 (45)	
Female	9 (60)	41 (55)	
**Age, years (IQR)**	12 (10–16)	15 (14–16)	0.01
**Indication for transplantation, n (%)**			0.003
Cholestasis/biliary atresia	6 (40)	61 (81)	
Metabolic diseases	2 (13)	5 (7)	
Liver tumors	3 (20)	5 (7)	
Cirrhosis (other)	4 (27)	3 (4)	
Severe acute liver failure	0 (0)	1 (1)	
**Transplant number, n (%)**			1.00
First	14 (93)	68 (91)	
Second	1 (7)	6 (8)	
Unknown	0 (0)	1 (1)	
**Type of transplantation, n (%)**			1.00
Hepatic	15 (100)	75 (100)	
Combined	0 (0)	0 (0)	
**Prior graft rejection, n (%)**			N/A
No	N/A	71 (95)	
Yes	N/A	4 (5)	
**Immunosuppressive treatment, n (%)**			N/A
TAC	N/A	27 (36)	
TAC+CE	N/A	39 (52)	
TAC+CE+MMF	N/A	2 (3)	
TAC+MMF	N/A	2 (3)	
CE+CS	N/A	5 (7)	
**Tacrolimus blood levels, ng/mL (IQR)**	N/A	3.80 (3.02–5.20)	N/A
**Cyclosporine blood levels, ng/mL (IQR)**	N/A	129.10 (49.30–314.50)	N/A
**FIB-4 index, (IQR)**	5.40 (2.01–8.72)	0.28 (0.20–0.45)	<0.001
**FIB-4 index-based liver fibrosis risk, n (%)**			<0.001
Unlikely	0 (0)	20 (27)	
Indeterminate	5 (33)	55 (73)	
Likely	10 (67)	0 (0)	

CE, corticosteroids; CS, cyclophosphamide; FIB-4, Fibrosis-4; IQR, interquartile range; MMF, mycophenolate mofetil; N/A, not applicable; TAC, tacrolimus.

The Pre-Tx cohort consisted of 15 patients, with similar sex distribution (40% males and 60% females). However, median age was significantly lower in the Pre-Tx cohort compared to Post-Tx (12 years, IQR [10-16] vs. 15 years IQR [14-16]; p=0.01). Although biliary atresia was the most common indication in both cohorts, it was markedly more frequent in the Post-Tx group (81% vs. 40%, p=0.003).

Biochemical and immunological parameters from Post-Tx and Pre-Tx cohorts are summarized in **Table 2**. As expected, liver function parameters were significantly altered in Pre-Tx patients (p<0.001) and, consequently, the FIB-4 index (**Table 1**) was higher in this group (5.40 IQR [2.01-8.72] vs. 0.28 IQR [0.20-0.45], p<0.001). Accordingly, 67% of Pre-Tx patients were likely to have liver fibrosis, compared with none in the Post-Tx group, whereas 27% of Post-Tx patients were unlikely to have liver fibrosis, compared with none in the Pre-Tx group (p<0.001).

**Table 2:** Biochemical and immunological parameters in two independent cohorts of pediatric liver transplant candidates (Pre-Tx) and long-term liver transplant recipients (Post-Tx).

Characteristics	Pre-Tx (n=15)	Post-Tx (n=75)	P-value
**Liver function parameters, IU/L (IQR)**			
Alanine Aminotransferase	631 (225–850)	25 (20–31)	<0.001
Aspartate Aminotransferase	1,210 (408–1,602)	19 (15–26)	<0.001
Gamma-Glutamyl Transferase	49 (38–111)	20 (15–35)	<0.001
Lactate Dehydrogenase	1,541 (725–2,029)	204 (181–231)	<0.001
Total Bilirubin	3.14 (1.78–3.83)	0.62 (0.45–0.86)	<0.001
Platelet Count (x10e3)	85 (58–149)	213 (168–258)	<0.001
**Immunoglobulins, mg/dL (IQR)**			
IgG	1,271 (1,015–1,451)	1,216 (1,015–1,361)	0.45
IgA	162 (107–215)	261 (188–330)	0.001
IgM	121 (100–162)	117 (88–159)	0.93
**ANA patterns by indirect immunofluorescence on HEp-2 cells, n (%)**			0.01
Not determined*	11 (73)	0 (0)	
Homogeneous	2 (50)	1 (1)	
Speckled	0 (0)	16 (21)	
Nucleolar	0 (0)	5 (7)	
Cytoplasmic	0 (0)	1 (1)	
Speckled + Homogeneous	0 (0)	1 (1)	
Speckled + Nucleolar	0 (0)	2 (3)	
Speckled + Cytoplasmic	1 (25)	1 (1)	
Nucleolar + Homogeneous	0 (0)	1 (1)	
Nucleolar + Cytoplasmic	0 (0)	1 (1)	
Negative	1 (25)	47 (63)	
**Autoantibody Patterns on Triple Rodent Tissue, n (%)**			0.10
Not determined*	11 (73)	0 (0)	
AMA	0 (0)	1 (1)	
LKM	0 (0)	0 (0)	
ASMA	0 (0)	0 (0)	
ACPA	1 (25)	0 (0)	
GSTT1	0 (0)	2 (3)	
Negative	3 (75)	72 (96)	
**Anti-HLA class-I antibodies, n (%)**	1 (8)	15 (20)	0.45
**Anti-HLA class-II antibodies, n (%)**	0 (0)	33 (44)	0.002
**Anti-MICA antibodies, n (%)**	1 (8)	10 (13)	1.00

ACPA, anti-parietal cell antibodies; AMA, anti-mitochondrial antibodies; ANA, anti-nuclear antibodies; ASMA, anti-smooth muscle antibodies; GSTT1, anti-glutathione S-transferase theta 1 antibodies; HLA, human leukocyte antigen; Ig, immunoglobulin; IQR, interquartile range; IU, international units; LKM, anti-liver-kidney microsomal antibodies; MICA, MHC class-I chain-related protein A; N/A, not applicable.

* “Not determined” cases are excluded from the pattern-specific percentage and p-value calculations.

Regarding immunoglobulins (**Table 2**), IgG and IgM levels were similar in both cohorts, but median IgA levels were significantly higher in the Post-Tx cohort compared to Pre-Tx patients (261 mg/dL IQR [188–330] vs. 162 mg/dL IQR [107–215], p=0.001).

### 3.2 Non-HLA antibody profiles and frequency

**Figure 1:**
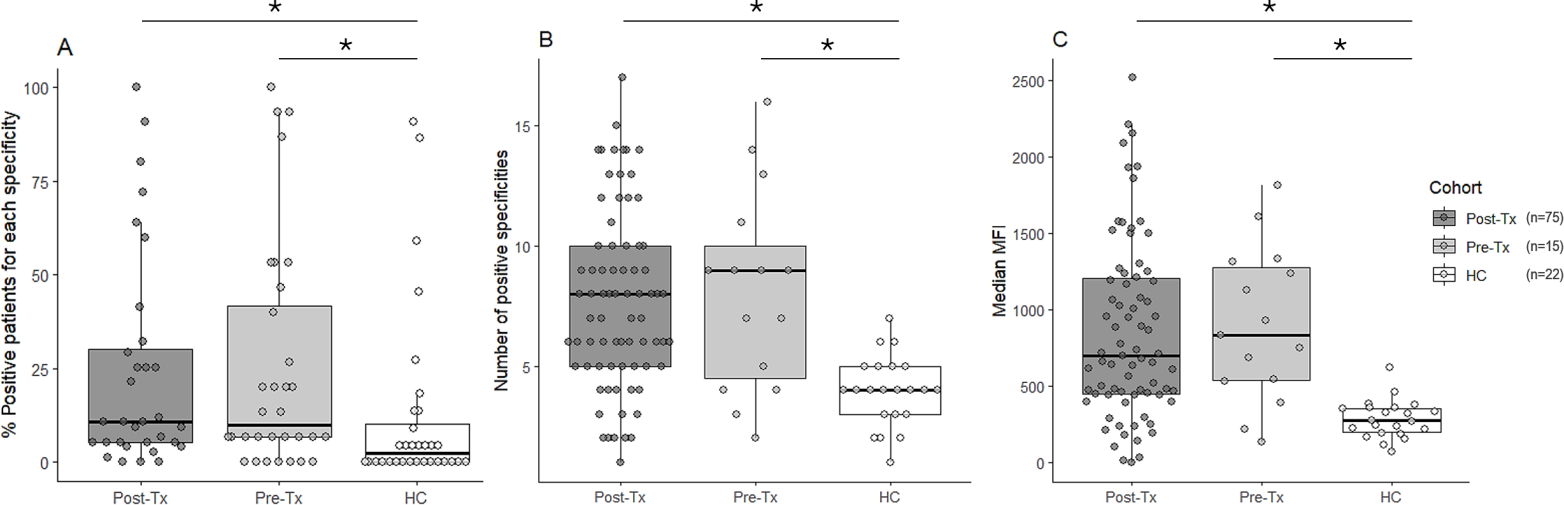
Frequency of positive patients for each non-HLA specificity **(A)**, number of positive non-HLA specificities per patient **(B)** and median mean fluorescence intensity (MFI) per patient **(C)** across three study cohorts: pediatric liver transplant recipients with long-term graft survival (Post-Tx) (n=75), pediatric patients on the waiting list for liver transplantation (Pre-Tx) (n=15) and adult healthy controls (HC) (n=22). Asterisks over horizontal lines indicate significant differences between the connected groups (p<0.05).

We first observed that both Post-Tx and Pre-Tx cohorts had a significantly increased frequency of patients positive for non-HLA antibodies (**Figure 1A**) compared to HC (Post-Tx 11% IQR [5–30] and Pre-Tx 10% IQR [7–72] vs. HC 2% IQR [0–10]; p=0.004 and p=0.005, respectively). That increment was also reflected in the number of positive specificities (**Figure 1B**), significantly higher in both patient cohorts compared to HC (Post-Tx 8 IQR [5–10] and Pre-Tx 9 IQR [5–10] vs. HC 4 IQR [3–5]; p<0.001 and p<0.001, respectively). Non-HLA antibody levels, assessed by MFI (**Figure 1C**), were markedly elevated in both Post-Tx and Pre-Tx compared to HC (Post-Tx 967 IQR [445–1,203] and Pre-Tx 833 IQR [537–1,278] vs. HC 273 IQR [200–354]; p<0.001 and p<0.001, respectively).

**Figure 2:**
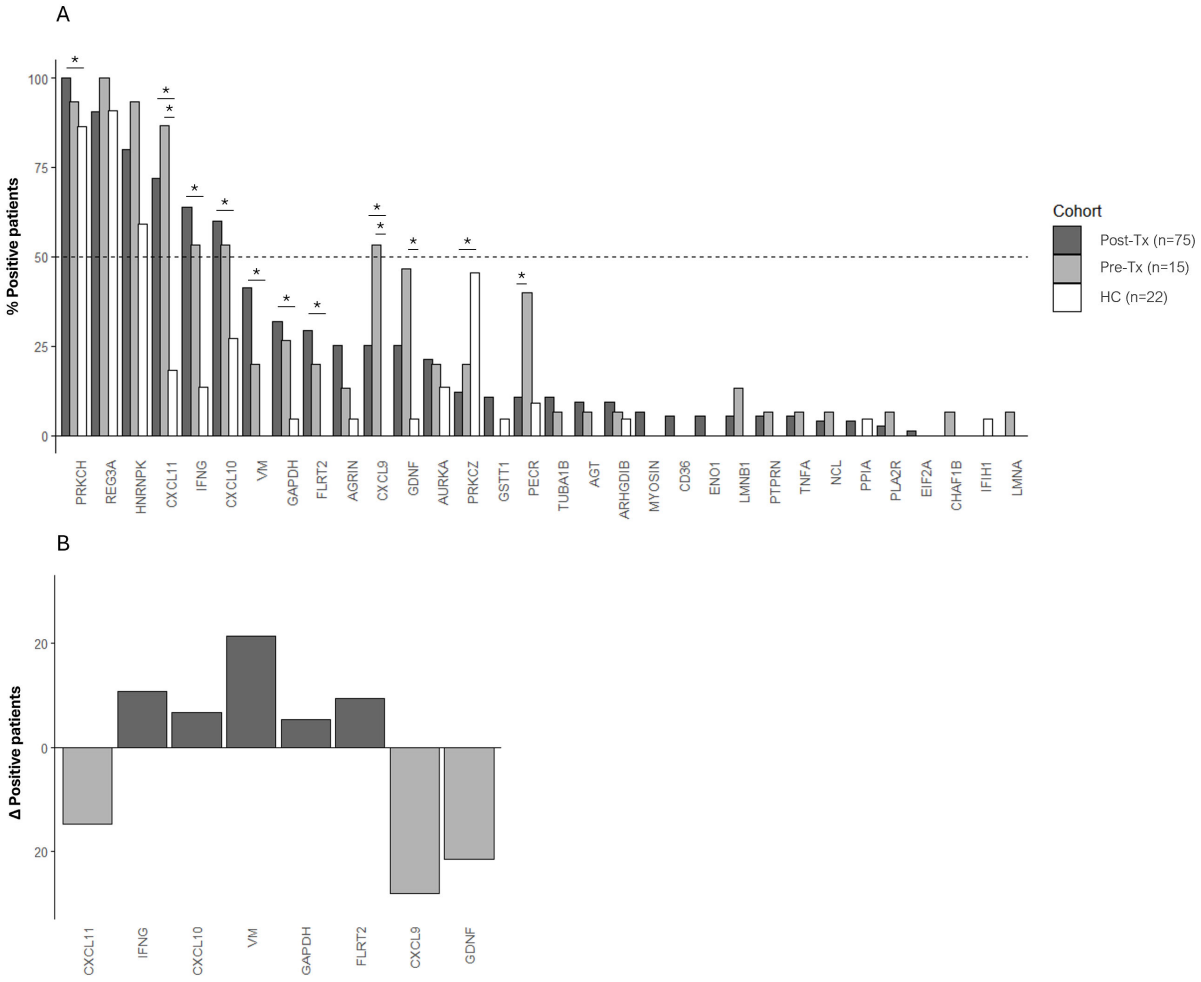
Frequency of positive patients for each non-HLA antibody specificity **(A)** in pediatric liver transplant recipients with long-term graft survival (Post-Tx) (n=75), pediatric patients on the waiting list for liver transplantation (Pre-Tx) (n=15) and adult healthy controls (HC) (n=22). The dashed horizontal line at 50% positivity denotes the exclusion threshold for high-frequency antibodies in HC. Asterisks over horizontal lines indicate significant differences between the connected groups (p<0.05). **(B)** Difference in prevalence between Post-Tx and Pre-Tx cohorts for the non-HLA specificities that showed significantly higher frequencies compared to HC. AGRIN, agrin; AGT angiotensinogen; ARHGDIB, Rho GDP-dissociation inhibitor 2; AURKA, aurora kinase A-interacting protein; CD36, platelet glycoprotein 4; CXCL9, C-X-C motif chemokine ligand 9; CXCL10, C-X-C motif chemokine ligand 10; CXCL11, C-X-C motif chemokine ligand 11; CHAF1B, chromatin assembly factor 1 subunit B; EIF2A, eukaryotic translation initiation factor 2A; ENO1, alpha-enolase; FLRT2, leucine-rich repeat transmembrane protein 2; GDNF, glial cell line-derived neurotrophic factor; GSTT1, glutathione S-transferase theta 1; GAPDH, glyceraldehyde-3-phosphate dehydrogenase; HNRNPK, heterogeneous nuclear ribonucleoprotein K; IFNG, interferon gamma; IFIH1, interferon-induced helicase C domain-containing protein 1; LMNA, lamin-A/C; LMNB, lamin-B1; MYOSIN, cardiac myosin-binding protein C; NCL, nucleolin; PPIA, peptidyl-prolyl cis-trans isomerase A; PECR, peroxisomal trans-2-enoyl-CoA reductase; PRKCH, protein kinase C eta; PRKCZ, protein kinase C zeta; PTPRN, receptor-type tyrosine-protein phosphatase-like N; REG3A, regenerating islet-derived protein 3-alpha; PLA2R, secretory phospholipase A2 receptor; TNFA, tumor necrosis factor alpha; TUBA1B, tubulin alpha-1B chain; VM, vimentin.

Next, we compared frequencies of positivity for each specificity (**Figure 2A**). Following the definition of high-frequency antibodies in healthy individuals from a previous study (20), we excluded from our analysis regenerating islet-derived protein 3-alpha (REG3A, 91%), protein kinase C eta (PRKCH, 86%) and HNRNPK (59%) specificities, that were present in more than 50% of HC in our cohort. Protein kinase C zeta (PRKCZ) was also excluded, as it was significantly increased in HC compared to Post-Tx (46% vs. 12%; p=0.004). Similarly, peroxisomal trans-2-enoyl-CoA reductase (PECR) was excluded, as no significant differences were observed between HC and either the Pre-Tx or Post-Tx cohorts; the only significant difference was between Pre-Tx and Post-Tx groups (40% vs. 11%; p=0.04).

We found eight specificities that were significantly increased in Post-Tx and/or Pre-Tx cohorts compared to HC: CXCL11, interferon gamma (IFNG), CXCL10, VM, glyceraldehyde-3-phosphate dehydrogenase (GAPDH), leucine-rich repeat transmembrane protein 2 (FLRT2), CXCL9 and glial cell line-derived neurotrophic factor (GDNF). These specificities were selected for further analysis based on both their statistical significance compared to HC and their relevance to processes associated with graft injury and inflammation. The most frequent in the Post-Tx group were IFNG (64% vs. 14%; p<0.001), CXCL10 (60% vs. 27%; p=0.03), VM (41% vs. 0%; p<0.001), GAPDH (32% vs. 5%; p=0.03) and FLRT2 (29% vs. 0%; p=0.007). These antibodies were also detected before transplantation, but their prevalence was notably higher in the Post-Tx cohort. The most pronounced difference between Pre-Tx and Post-Tx was observed for anti-VM antibodies (**Figure 2B**), with a prevalence increase of 21% in the Post-Tx group relative to the Pre-Tx group.

On the other hand, anti-CXCL11 and anti-CXCL9 antibodies were more frequent in Pre-Tx patients (87% and 53%, respectively), although they were also significantly increased in Post-Tx individuals compared to HC (CXCL11 72% vs. 18%, p<0.001; CXCL9 25% vs. 0%, p=0.02). Interestingly, anti-GDNF antibodies were significantly elevated only in the Pre-Tx group compared to HC (47% vs. 5%; p=0.02). Those three specificities (CXCL11, CXCL9 and GDNF) showed a higher prevalence increase in the Pre-Tx cohort compared to Post-Tx group (**Figure 2B**).

### 3.3 Non-HLA antibody intensity and specificity clustering

**Figure 3:**
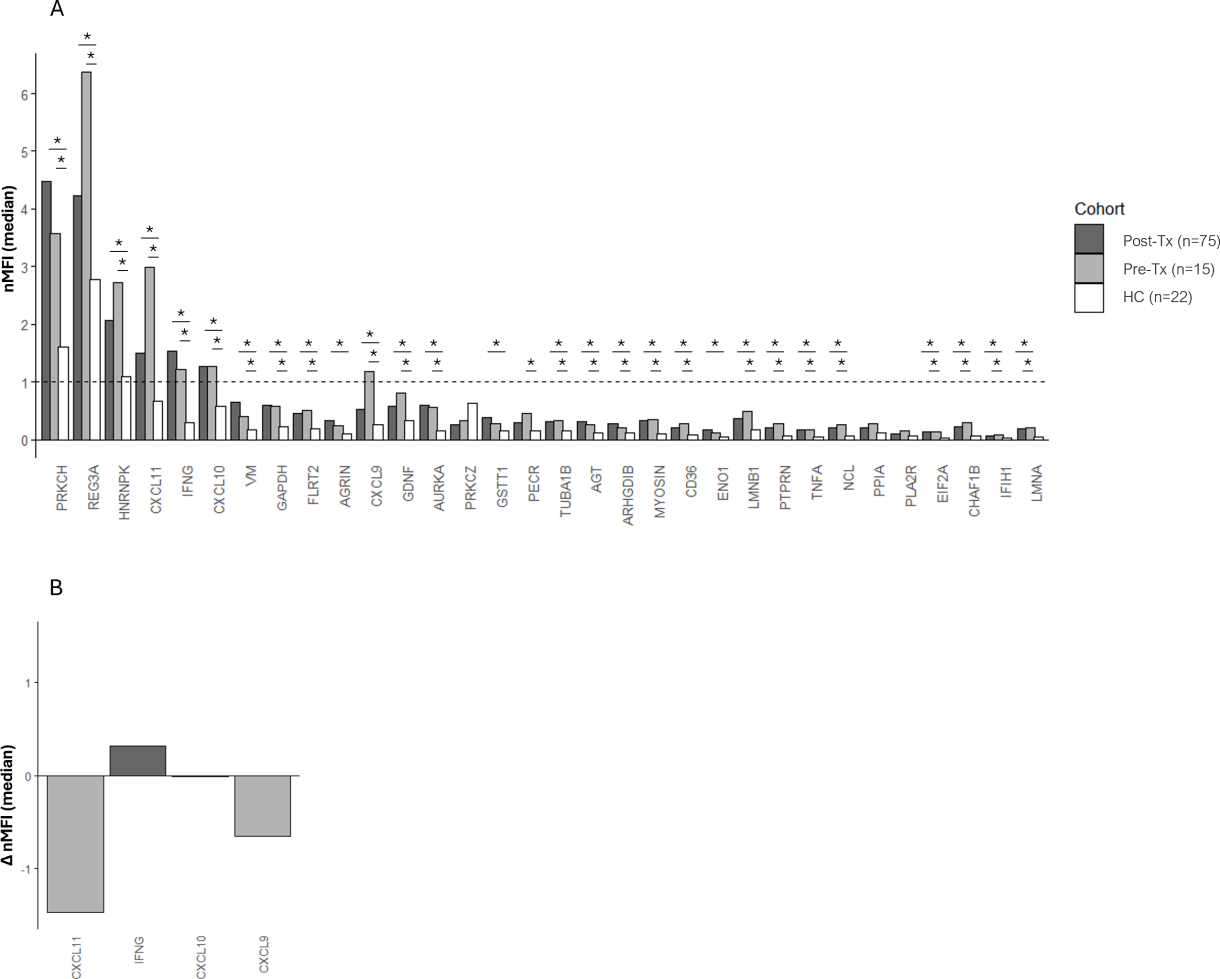
Median of the normalized mean fluorescence intensity (nMFI) from each non-HLA antibody specificity **(A)** in pediatric liver transplant recipients with more than ten years of follow-up (Post-Tx) (n=75), pediatric patients on the waiting list for liver transplantation (Pre-Tx) (n=15) and adult healthy controls (HC) (n=22). The dashed horizontal line at nMFI 1 denotes the positivity cutoff. Asterisks over horizontal lines indicate significant differences between the connected groups (p<0.05). **(B)** Difference in the nMFI between Post-Tx and Pre-Tx cohorts for positive non-HLA specificities that were significantly higher compared to HC. AGRIN, agrin; AGT angiotensinogen; ARHGDIB, Rho GDP-dissociation inhibitor 2; AURKA, aurora kinase A-interacting protein; CD36, platelet glycoprotein 4; CXCL9, C-X-C motif chemokine ligand 9; CXCL10, C-X-C motif chemokine ligand 10; CXCL11, C-X-C motif chemokine ligand 11; CHAF1B, chromatin assembly factor 1 subunit B; EIF2A, eukaryotic translation initiation factor 2A; ENO1, alpha-enolase; FLRT2, leucine-rich repeat transmembrane protein 2; GDNF, glial cell line-derived neurotrophic factor; GSTT1, glutathione S-transferase theta 1; GAPDH, glyceraldehyde-3-phosphate dehydrogenase; HNRNPK, heterogeneous nuclear ribonucleoprotein K; IFNG, interferon gamma; IFIH1, interferon-induced helicase C domain-containing protein 1; LMNA, lamin-A/C; LMNB, lamin-B1; MYOSIN, cardiac myosin-binding protein C; NCL, nucleolin; PPIA, peptidyl-prolyl cis-trans isomerase A; PECR, peroxisomal trans-2-enoyl-CoA reductase; PRKCH, protein kinase C eta; PRKCZ, protein kinase C zeta; PTPRN, receptor-type tyrosine-protein phosphatase-like N; REG3A, regenerating islet-derived protein 3-alpha; PLA2R, secretory phospholipase A2 receptor; TNFA, tumor necrosis factor alpha; TUBA1B, tubulin alpha-1B chain; VM, vimentin.

We next analyzed the nMFI for each specificity across the three cohorts, excluding REG3A, PRKCH and HNRNPK, previously removed for high frequency in HC and now also for elevated nMFI in that group (**Figure 3A**). In the Post-Tx cohort, three specificities showed a nMFI >1 and were significantly elevated compared to HC: IFNG (1.54 IQR [0.70–3.11] vs. 0.29 IQR [0.19–0.64]; p<0.001), CXCL11 (1.51 IQR [0.95–2.44] vs. 0.66 IQR [0.42–0.93]; p<0.001) and CXCL10 (1.27 IQR [0.54–2.33] vs. 0.58 IQR [0.37–1.03]; p=0.01). Those same specificities also exhibited significantly higher nMFI values in the Pre-Tx cohort compared to HC. Additionally, CXCL9 reached a nMFI >1 in the Pre-Tx group (1.18 IQR [0.36–1.74]) and, although its nMFI was below this threshold in Post-Tx (0.53 IQR [0.25–0.99]), it remained significantly elevated compared to HC (p=0.03).

**Figure 3B** depicts the change in nMFI (ΔnMFI) between Pre-Tx and Post-Tx for each specificity. Anti-IFNG antibodies showed a positive ΔnMFI, indicating an increase in intensity following transplantation. In contrast, anti-CXCL11, anti-CXCL9 and anti-CXCL10 antibodies showed negative values, reflecting a decrease in antibody levels post-transplantation. All positivity rates and nMFI values for each specificity are detailed on **Supplementary Tables S1**, **S2**.

**Figure 4:**
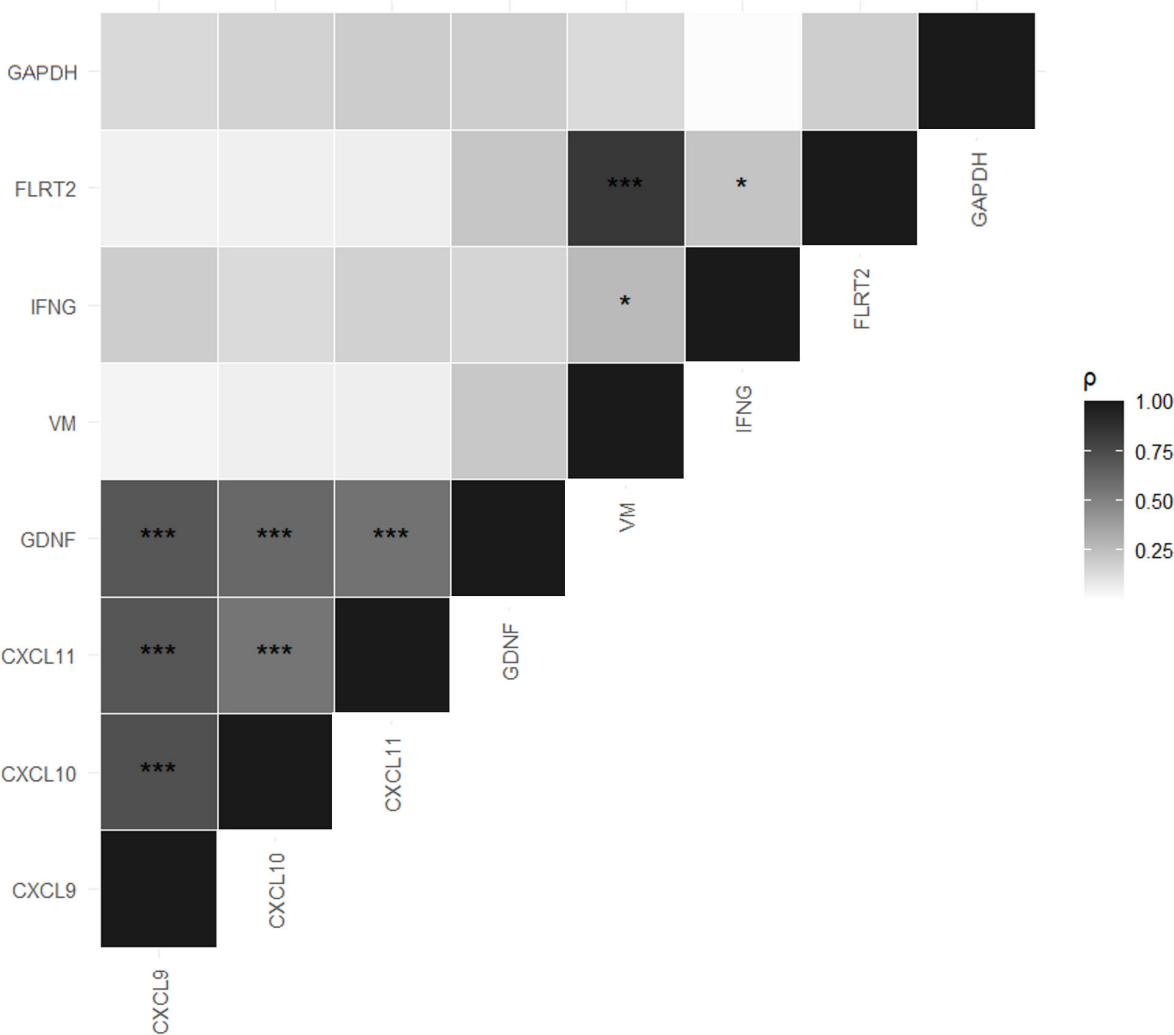
Heatmap representing the correlation matrix of the normalized mean fluorescence intensity values of non-HLA antibodies that showed significantly higher frequencies compared to healthy controls (HC). Darker tones indicate stronger positive correlations and lighter tones indicate weaker or negative correlations. *p<0.05; **p<0.01; ***p<0.001. CXCL9, C-X-C motif chemokine ligand 9; CXCL10, C-X-C motif chemokine ligand 10; CXCL11, C-X-C motif chemokine ligand 11; FLRT2, leucine-rich repeat transmembrane protein 2; GDNF, glial cell line-derived neurotrophic factor; GAPDH, glyceraldehyde-3-phosphate dehydrogenase; IFNG, interferon gamma; VM, vimentin.

Lastly, we analyzed correlations among specificities significantly increased in Pre-Tx or Post-Tx versus HC, revealing two distinct clusters. The first group, comprising VM, FLRT2 and IFNG, showed positive correlations (ρ=0.23-0.84, p<0.05). The second cluster, consisting of GDNF, CXCL9, CXCL10 and CXCL11, also demonstrated significant intercorrelations (ρ=0.56-0.72, p<0.001). These patterns were visually represented in a heatmap (**Figure 4**).

### 3.4 Non-HLA antibody positivity in relation to alloimmune and autoimmune responses

We next compared alloimmune and autoimmune responses between LTx candidates and long-term recipients. To assess autoimmune responses, we first evaluated ANA patterns (**Table 2**), which differed significantly between groups (p=0.01). Notably, this analysis was not performed in 73% of Pre-Tx patients, limiting pre-transplant characterization. Among those tested, the homogeneous ANA pattern was the most frequent (50%), while 25% cases were negative. In Post-Tx recipients, ANA was assessed in all cases, with 63% testing negative. Among ANA-positive Post-Tx patients, the speckled pattern predominated (21%). Using triple rodent tissue, 75% of tested PreTx patients were negative and 25% positive for ACPA. In Post-Tx recipients, 96% were negative, with isolated cases of AMA (1%) and anti-GSTT1 (3%).

To characterize alloimmune responses, we evaluated the presence of anti-HLA class-I/II and anti-MICA antibodies in both patient cohorts (**Table 2**). In the Pre-Tx group, one (8%) patient tested positive for anti-class-I HLA and one (8%) for anti-MICA antibodies. In contrast, the Post-Tx group showed higher frequencies of alloantibody positivity: 15 (20%) patients were positive for anti-HLA class-I, 33 (44%) for anti-HLA class-II and 10 (13%) for anti-MICA antibodies. The prevalence of anti-HLA class-II antibodies post-transplantation was significantly higher (p=0.002).

Furthermore, we examined associations between antibodies in the Post-Tx cohort (**Table 3**). Although no significant correlations were found, non-HLA antibodies were more frequent in patients positive for anti-HLA class-II antibodies compared to anti-HLA class-I-positive patients (43% vs. 19%, p=0.63). Non-HLA antibodies were also present in MICA-positive patients, but overall non-HLA positivity was higher in MICA-negative individuals (81% vs. 13%, p=1.00). Interestingly, ANA positivity was common in non-HLA-positive patients (36%, p=1.00), whereas AMA antibodies were absent (p=0.05).

**Table 3:** Distribution of allo- and autoantibodies in a cohort of pediatric long-term liver transplant recipients, stratified by non-HLA antibody status.

	Non-HLA negative, n (%)	Non-HLA positive, n (%)	Total, n (%)	P-values
Anti-HLA class-I antibodies				1.00
Negative	3 (4)	57 (76)	**60 (80)**	
Positive	1 (1)	14 (19)	**15 (20)**	
**Total, n (%)**	**4 (5)**	**71 (95)**	**75 (100)**	
Anti-HLA class-II antibodies				0.63
Negative	3 (3)	39 (52)	**42 (56)**	
Positive	1 (1)	32 (43)	**33 (44)**	
**Total, n (%)**	**4 (5)**	**71 (95)**	**75 (100)**	
Anti-MICA antibodies				1.00
Negative	4 (5)	61 (81)	**65 (87)**	
Positive	0 (0)	10 (13)	**10 (13)**	
**Total, n (%)**	**4 (5)**	**71 (95)**	**75 (100)**	
Anti-nuclear antibodies				1.00
Negative	3 (4)	44 (59)	**47 (63)**	
Positive	1 (1)	27 (36)	**28 (37)**	
**Total, n (%)**	**4 (5)**	**71 (95)**	**75 (100)**	
Anti-mitochondrial antibodies				0.05
Negative	3 (4)	71 (95)	**74 (99)**	
Positive	1 (1)	0 (0)	**1 (1)**	
**Total, n (%)**	**4 (5)**	**71 (95)**	**75 (100)**	

MICA, MHC class-I chain-related protein A; HLA, human leukocyte antigen.

### 3.5 Non-HLA and anti-HLA antibody associations with post-transplant FIB-4 index

**Figure 5:**
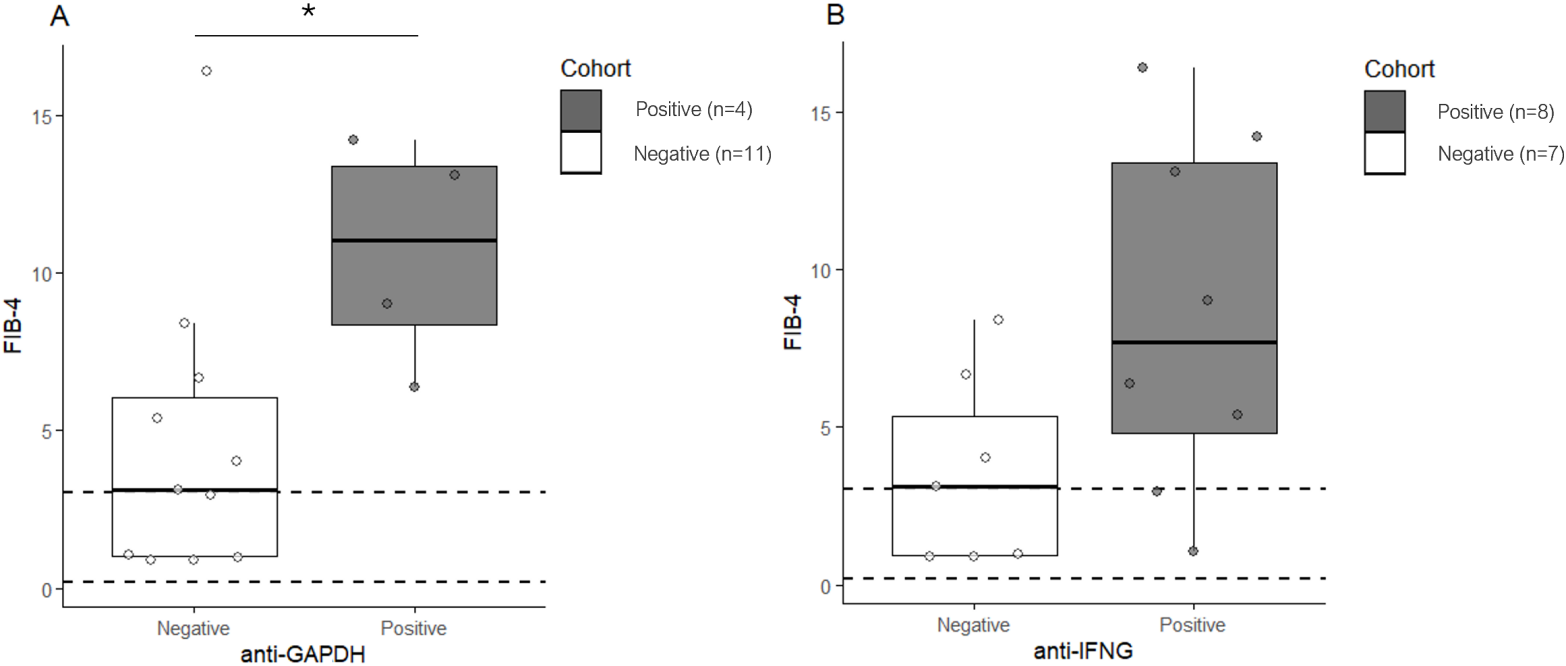
Fibrosis-4 index (FIB-4) values in a cohort of pediatric patients on the waiting list for liver transplantation (Pre-Tx cohort) (n=15) segregated according to **(A)** anti-glyceraldehyde-3-phosphate dehydrogenase (GAPDH) antibody positivity (n=4 positive, n=11 negative) and **(B)** anti-interferon gamma (IFNG) antibody positivity (n=8 positive, n=7 negative). The dashed horizontal lines indicate the predefined FIB-4 thresholds at 0.20 and 3.03, used to classify the risk of liver fibrosis as unlikely (<0.20), indeterminate (0.20–3.03) or likely (≥3.03). These thresholds represent operational cut-offs derived from pediatric transplantation data for risk categorization and do not serve as definitive diagnostic criteria. Asterisks over horizontal lines indicate significant differences (p<0.05).

To explore potential associations between immune reactivity and liver fibrosis risk, we compared median FIB-4 values according to non-HLA antibody status. In the Pre-Tx cohort, FIB-4 index was significantly higher in patients positive for anti-GAPDH antibodies (**Figure 5A**; 11.06 IQR [8.36–13.38] vs. 3.12 IQR [1.01–6.04], p=0.04). Patients positive for anti-IFNG antibodies also showed higher FIB-4 values, although differences did not reach statistical significance (**Figure 5B**; 7.71 IQR [4.79–13.38] vs. 3.12 IQR [0.94–5.36], p=0.07).

**Figure 6:**
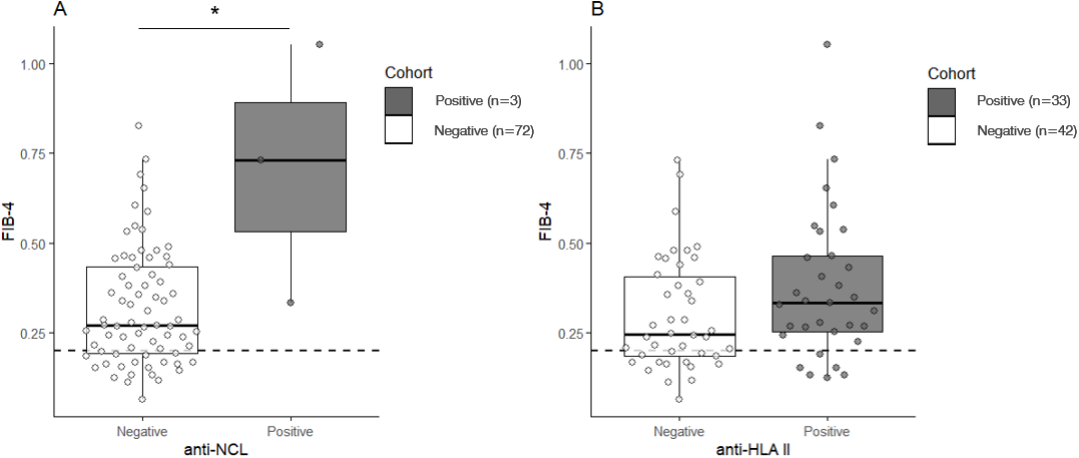
Fibrosis-4 index (FIB-4) values in a cohort of pediatric liver transplant recipients with long-term graft survival (Post-Tx cohort) (n=75) segregated according to **(A)** anti-nucleolin (NCL) antibody positivity (n=3 positive, n=72 negative) and **(B)** anti-HLA class-II antibody positivity (n=33 positive, n=42 negative). The dashed horizontal line indicates the FIB-4 threshold of 0.20, a cutoff above which liver fibrosis cannot be ruled out. This threshold represents an operational cut-off derived from pediatric transplantation data for risk categorization and does not serve as a definitive diagnostic criterion. Asterisks over horizontal lines indicate significant differences (p<0.05).

In the Post-Tx cohort, FIB-4 values were significantly increased in patients positive for anti-nucleolin (NCL) antibodies compared to those negative (**Figure 6A**; 0.73 IQR [0.53–0.89] vs. 0.27 IQR [0.19–0.43], p=0.04). Post-Tx patients positive for anti-HLA class-II antibodies also showed higher FIB-4 values than negative patients, but that difference did not reach statistical significance (**Figure 6B**; 0.33 IQR [0.25–0.46] vs. 0.24 IQR [0.19–0.41], p=0.09).

We next examined differences in clinical and immunological variables between patients with and without fibrosis risk. Since no Post-Tx patients exceeded a FIB-4 value of 3.03, the category of “likely fibrosis” was excluded from the analysis. Consequently, a FIB-4 index ≥0.20 was adopted as an interpretative threshold to retain discriminatory power in our analysis. It is important to note that this redefinition does not imply that patients with a FIB-4 index ≥0.20 have advanced fibrosis but rather reflects a level above which fibrosis cannot be ruled out based on established pediatric thresholds. Interestingly, sex distribution differed significantly in relation to FIB-4 values (**Table 4**), with males more frequently presenting scores ≥0.20 (53% vs. 25%, p=0.04). Among the eight non-HLA antibody specificities significantly elevated versus HC, no differences in antibody positivity were observed between FIB-4 index groups (**Table 4**).

**Table 4:** Epidemiologic and clinical features in a cohort of long-term liver transplant recipients (Post-Tx) stratified by their FIB-4 values.

Characteristics	FIB-4 index <0.2 (n=20)	FIB-4 index ≥0.2 (n=55)	P-value
**Sex, n (%)**			0.04
Male	5 (25)	29 (53)	
Female	15 (75)	26 (47)	
**Age, n (%)**			0.20
>18 years	2 (10)	5 (9)	
12–18 years	15 (75)	48 (87)	
6–12 years	3 (15)	2 (4)	
**Indication for transplantation, n (%)**			0.24
Cholestasis/biliary atresia	16 (80)	45 (82)	
Metabolic diseases	3 (15)	2 (4)	
Liver tumors	0 (0)	5 (9)	
Cirrhosis (other)	1 (5)	2 (4)	
Severe acute liver failure	0 (0)	1 (2)	
**Transplant number, n (%)**			0.51
First	17 (85)	51 (93)	
Second	3 (15)	3 (5)	
Unknown	0 (0)	1 (2)	
**Prior graft rejection, n (%)**			0.29
No	18 (90)	53 (96)	
Yes	2 (10)	2 (4)	
**Immunosuppressive treatment, n (%)**			0.88
TAC	8 (40)	19 (35)	
TAC+CE	10 (50)	29 (53)	
TAC+CE+MMF	0 (0)	2 (4)	
TAC+MMF	1 (5)	1 (2)	
CE+CS	1 (5)	4 (7)	
**Anti-VM antibodies, n (%)**			0.68
Positive	7 (35)	24 (44)	
Negative	13 (65)	31 (56)	
**Anti-IFNG antibodies, n (%)**			0.21
Positive	10 (50)	38 (69)	
Negative	10 (50)	17 (31)	
**Anti-FLRT2 antibodies, n (%)**			0.15
Positive	3 (15)	19 (35)	
Negative	17 (85)	36 (65)	
**Anti-CXCL11 antibodies, n (%)**			0.27
Positive	12 (60)	42 (76)	
Negative	8 (40)	13 (24)	
**Anti-CXCL10 antibodies, n (%)**			0.79
Positive	11 (55)	34 (62)	
Negative	9 (45)	21 (38)	
**Anti-CXCL9 antibodies, n (%)**			0.79
Positive	6 (30)	13 (24)	
Negative	14 (70)	42 (76)	
**Anti-GAPDH antibodies, n (%)**			1.00
Positive	6 (30)	18 (33)	
Negative	14 (70)	37 (67)	
**Anti-GDNF antibodies, n (%)**			0.08
Positive	2 (10)	17 (31)	
Negative	18 (90)	38 (69)	
**Anti-HLA class-I antibodies, n (%)**			0.75
Positive	3 (15)	12 (22)	
Negative	17 (85)	43 (78)	
**Anti-HLA class-II antibodies, n (%)**			0.23
Positive	6 (30)	27 (49)	
Negative	14 (70)	28 (51)	
**Anti-MICA antibodies, n (%)**			0.05
Positive	0 (0)	10 (18)	
Negative	20 (100)	45 (82)	

CE, corticosteroids; CS, cyclophosphamide; FIB-4, Fibrosis-4; FLRT2, leucine-rich repeat transmembrane protein 2; GAPDH, glyceraldehyde-3-phosphate dehydrogenase; GDNF, glial cell line-derived neurotrophic factor; HLA, human leukocyte antigen; IFNG, interferon gamma; IQR, interquartile range; MICA, MHC class-I chain-related protein A; MMF, mycophenolate mofetil; TAC, tacrolimus; VM, vimentin.

**Figure 7:**
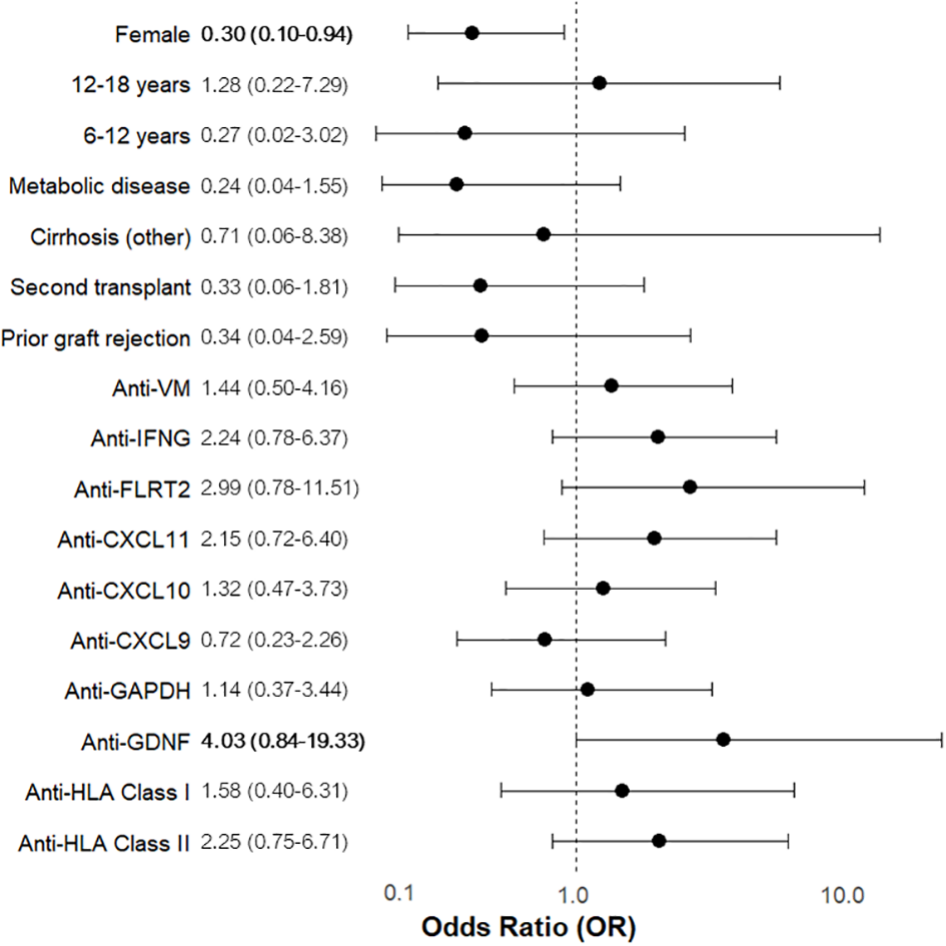
Forest plot representing the results of univariate logistic regression analyses assessing the association between clinical variables, HLA antibody positivity and non-HLA antibody positivity with the likelihood of having a Fibrosis-4 value ≥0.2 (fibrosis not excluded) in pediatric liver transplant recipients with long-term graft survival (Post-Tx cohort, n=75). Odds ratios (OR) are shown with 95% confidence intervals. Variables with a p-value<0.1 are marked in bold. Variables for which the model could not estimate an OR due to the absence of events in one of the comparison groups were excluded from the analysis. CXCL9, C-X-C motif chemokine ligand 9; CXCL10, C-X-C motif chemokine ligand 10; CXCL11, C-X-C motif chemokine ligand 11; FLRT2, leucine-rich repeat transmembrane protein 2; GDNF, glial cell line-derived neurotrophic factor; GAPDH, glyceraldehyde-3-phosphate dehydrogenase; IFNG, interferon gamma; VM, vimentin.

In the univariate analysis (**Figure 7**), female patients had significantly lower odds of presenting FIB-4 index ≥0.20 (odds ratio [OR] 0.30 [0.09-0.89], p=0.04). In contrast, anti-GDNF antibody positivity showed a trend toward an increased risk of elevated FIB-4 index in the univariate analysis (OR 4.03 [1.00-27.13], p=0.08). In the group with FIB-4 index ≥0.20, 31% (17/55) of patients were positive for anti-GDNF, compared to 10% (2/20) in the FIB-4 index <0.20 group. The multivariate model revealed that both female sex and anti-GDNF antibody positivity were independently associated with FIB-4 index elevation. Female sex remained protective (OR 0.23 [0.06-0.72], p=0.02), whereas anti-GDNF antibody positivity was independently associated with increased risk of elevated FIB-4 index (OR 5.61 [1.32-39.30], p=0.04).

## 4 Discussion

This study assessed the prevalence of non-HLA specificities and their association with liver fibrosis risk in pediatric recipients >10 years post-LTx, compared with waitlisted children and healthy adult controls. Both Post-Tx and Pre-Tx cohorts showed significantly higher prevalence of eight non-HLA antibodies versus HC, and anti-GDNF positivity was independently associated with higher FIB-4 values in our cross-sectional analysis. These findings should be considered as preliminary, pointing to a possible role of anti-GDNF as a biomarker of liver injury.

We first examined the overall non-HLA antibody response by frequency, MFI and number of positive specificities. Although no significant differences were observed between Pre-Tx and Post-Tx groups, MFI values tended to be lower post-transplantation, likely due to immunosuppression (34). Both patient cohorts showed higher positivity rates, number of specificities and MFI than HC. Differences in age (children vs. adults) and sample type (serum vs. plasma) may have influenced these findings. Previous studies using LABScreen™ Autoantibody have focused on transplant-related outcomes rather than age-related differences (35), so no evidence suggests that higher positivity in our pediatric cohorts is age-driven. Nonetheless, age-matched pediatric HC would help clarify this point.

Regarding sample type, LABScreen™ Autoantibody assay is recommended for serum, as ethylenediaminetetra-acetic acid (EDTA) treatment can increase background signal per manufacturer guidelines. However, plasma samples in our HC cohort were derived from heparin-treated blood, which may improve sensitivity for low-concentration analytes (36). Despite this, HC samples showed lower signal intensity than patient samples, suggesting that the reduced median values likely reflect true biological differences.

In our analysis, autoantibodies against REG3A, PRKCH and HNRNPK were excluded, due to their high prevalence in HC. Similarly, a recent study in 92 healthy individuals reported comparable rates: 87%, 85% and 70% for REG3A, PRKCH and HNRNPK, respectively (37). Anti-IFNG was also frequently detected in that study (87%), but in our cohort this antibody was not markedly increased in HC. We also found that anti-PECR antibodies were more prevalent in Pre-Tx than Post-Tx patients. Dinavahi et al. (38) reported in 49 KTx recipients that pre-transplant anti-PECR antibodies were associated with glomerulopathy, but found no differences between pre- and post-transplant sera. This discrepancy may reflect methodological differences, as they used a commercial ELISA instead of a Luminex-based assay.

On the other hand, CXCL9, CXCL10, CXCL11, GAPDH, GDNF, FLRT2, IFNG and VM specificities were significantly increased in Pre-Tx and/or Post-Tx patients versus HC. To our knowledge, only two studies have examined non-HLA antibodies in LTx. In the first one, 93 adult re-transplant recipients were tested for 33 non-HLA antibodies by LABScreen™ Autoantibody assay. Antibodies against agrin (AGRIN), chromatin assembly factor 1 subunit B (CHAF1B) and LG3 were associated with graft loss, but only anti-LG3 remained significant in the multivariate model (26). Interestingly, they only assessed AGRIN using the 95^th^ percentile as cut-off, and LG3 was not included in our study, which may explain the lack of replication. As a limitation, our analysis did not include other non-HLA specificities previously associated with fibrosis, such as collagen, LG3, AT1R and ETAR (26–28).

In the second study, 101 pediatric LTx recipients were screened for 60 non-HLA antibodies using LIFECODES™ antibody test. Small nuclear ribonucleoprotein polypeptides B (SNRPB), GSTT1 and actin were associated with biopsy-proven acute cellular rejection (39). Differences from our findings may reflect differences in sample timing (median 382 days post-transplant) and assay-related variability between LABScreen™ and LIFECODES™, which have distinct associations with rejection or graft loss (34, 40).

Evidence from other solid-organ transplants supports a role for non-HLA antibodies in graft injury. Anti-GAPDH antibodies have been associated with chronic lung-allograft dysfunction (21) and cardiac-allograft rejection (24, 25, 41). Similarly, cardiac rejection has been also linked to anti-CXCL11 antibodies (41), whereas KTx recipients exhibited elevated anti-CXCL10 and anti-CXCL11 antibodies during early graft rejection (34). Notably, increasing urinary CXCL9 and CXCL10 levels have been proposed as non-invasive markers of KTx rejection (42, 43). CXCL9 and CXCL10 are also increased in LTx recipients with early graft dysfunction (44) and patients with chronic hepatitis B or C and liver injury (45, 46). Although antibodies to these chemokines have not yet been linked to liver fibrosis risk, our findings suggest a possible interplay between cytokine expression and autoantibody development.

Based on nMFI, two clusters were identified: one comprising VM, FLRT2 and IFNG; and another including GDNF, CXCL9, CXCL10 and CXCL11. These results partially align with Alhamdan et al. (35), who analyzed non-HLA antibodies in 25 lung and 13 KTx recipients. They reported three clusters: fibrotic products (fibronectin and collagens I-V), cytoskeletal proteins (including VM and FLRT2) and signaling molecules (including IFNG, GDNF, CXCL10 and CXCL11), overlapping with ours.

Regarding HLA-DSA, they have been linked to progressive LTx fibrosis (15, 16). However, we found no significant association between anti-HLA class-I/II antibodies and elevated FIB-4 index. This limitation likely reflects the impossibility to identify donor-specificity, as only a general anti-HLA screening assay was available. Nonetheless, anti-HLA class-II positivity was significantly higher in Post-Tx than Pre-Tx samples, indicating alloimmunization triggered by transplantation.

Remarkably, anti-GDNF antibodies were independently associated with elevated FIB-4 values in the multivariate analysis. To our knowledge, this is the first study linking these antibodies to FIB-4 index. This raises the possibility that GDNF overexpression may induce corresponding autoantibodies, particularly in the context of liver injury or fibrosis. However, this hypothesis remains speculative and cannot be addressed within the scope of the present study due to the absence of paired tissue data. In contrast, previous evidence has already established that GDNF levels increase with fibrosis severity. In a recent study, Tao et al. (47) reported progressively higher GDNF levels with increasing fibrosis severity in 239 patients compared to HC. Moreover, serum GDNF outperformed FIB-4 values in distinguishing stage F4 from F3 fibrosis in chronic hepatitis B (48).

We acknowledge the limitation of using the FIB-4 index rather than histology, which may reduce sensitivity for detecting subtle fibrotic changes. Furthermore, it is important to note that FIB-4 index is a surrogate marker with limited validation in pediatric transplant populations, and its application in the current study is restricted by the absence of direct biopsy correlation. This study has additional limitations. Although donor age is a known factor associated with graft fibrosis (49, 50), it was not available in our database and could not be evaluated. Moreover, beyond the limited sample size, which may have constrained the statistical power of the analyses, the absence of donor HLA typing prevented the identification of HLA-DSA. Consequently, our findings regarding alloimmunity reflect general anti-HLA class I and class II reactivity rather than confirmed donor-specific responses.

Furthermore, the lack of paired pre- and post-transplant samples prevented distinguishing preformed from *de novo* antibodies. To partially mitigate this limitation, a separate Pre-Tx cohort was included as a baseline for pre-transplant reactivity. However, the cross-sectional design of the study inherently limits our ability to establish causal inferences. Nonetheless, the detection of non-HLA antibodies before transplantation likely reflects underlying liver injury rather than a causal role in graft rejection. This is supported by Kang et al. (51), who reported that positive pre-transplant crossmatches without HLA-DSA are often explained by non-HLA antibodies. Finally, the wide confidence intervals observed for some associations, particularly those involving anti-GDNF antibody positivity, indicate statistical imprecision due to the limited number of events and highlight the need for cautious interpretation.

In conclusion, our study demonstrates that eight non-HLA antibodies are more prevalent in both pediatric LTx recipients and waitlisted candidates compared to HC. Among these, anti-GDNF antibodies were independently associated with higher FIB-4 values in our cross-sectional analysis. These findings are exploratory and warrant further confirmation in larger, ideally longitudinal cohorts with histological endpoints to clarify the potential role of these antibodies as biomarkers for liver injury.

## Data Availability

The original contributions presented in the study are included in the article/**Supplementary Material**. Further inquiries can be directed to the corresponding author.
