# Supplementary material for: Prevalence of non-HLA antibodies and their association with FIB-4 index-based fibrosis risk in pediatric liver transplant recipients with long-term graft survival (>10 years)
Source: Front Immunol. 2026 Jun 15;17:1813802. doi: 10.3389/fimmu.2026.1813802 (PMC13310716; doi:10.3389/fimmu.2026.1813802)
Supplement: Supplementary file 1 [file SupplementaryFile1.docx]

Supplementary Material

# Supplementary tables

**Table S1. Positivity rates of 32 non-HLA antibodies in three independent cohorts: adult healthy controls (HC, n=22), pediatric liver transplant candidates (Pre-Tx, n=15) and long-term liver transplant recipients (Post-Tx, n=75). The change in prevalence (∆%) for each specificity was calculated by subtracting the frequency of positives from Pre-Tx to the frequency of positives from Post-Tx.**

| **Antibody specificity** | **HC, n (%)** | **Pre-Tx, n (%)** | **Post-Tx, n (%)** | **∆%** | **P-value** |
| --- | --- | --- | --- | --- | --- |
| AGRIN | 1 (5) | 2 (13) | 19 (25) | 12 | 0.08 |
| AGT | 0 (0) | 1 (7) | 7 (9) | 2 | 0.46 |
| ARHGDIB | 1 (5) | 1 (7) | 7 (9) | 2 | 0.88 |
| AURKA | 3 (14) | 3 (20) | 16 (21) | 1 | 0.77 |
| CD36 | 0 (0) | 0 (0) | 4 (5) | 5 | 0.76 |
| CHAF1B | 0 (0) | 1 (7) | 0 (0) | -7 | 0.13 |
| CXCL10 | 6 (27) | 8 (53) | 45 (60) | 7 | 0.02 |
| CXCL11 | 4 (18) | 13 (87) | 54 (72) | -15 | <0.001 |
| CXCL9 | 0 (0) | 8 (53) | 19 (25) | -28 | <0.001 |
| EIF2A | 0 (0) | 0 (0) | 1 (1) | 1 | 1.00 |
| ENO1 | 0 (0) | 0 (0) | 4 (5) | 5 | 0.76 |
| FLRT2 | 0 (0) | 3 (20) | 22 (29) | 9 | 0.005 |
| GADPH | 1 (5) | 4 (27) | 24 (32) | 5 | 0.02 |
| GDNF | 1 (5) | 7 (47) | 19 (25) | -22 | 0.008 |
| GSTT1 | 1 (5) | 0 (0) | 8 (11) | 11 | 0.43 |
| HNRNPK | 13 (59) | 14 (93) | 60 (80) | -13 | 0.04 |
| IFIH1 | 1 (5) | 0 (0) | 0 (0) | 0 | 0.33 |
| IFNG | 3 (14) | 8 (53) | 48 (64) | 11 | <0.001 |
| LMNA | 0 (0) | 1 (7) | 0 (0) | -7 | 0.13 |
| LMNB1 | 0 (0) | 2 (13) | 4 (5) | -8 | 0.19 |
| MYOSIN | 0 (0) | 0 (0) | 5 (7) | 7 | 0.5 |
| NCL | 0 (0) | 1 (7) | 3 (4) | -3 | 0.56 |
| PECR | 2 (9) | 6 (40) | 8 (11) | -29 | 0.01 |
| PLA2R | 0 (0) | 1 (7) | 2 (3) | -4 | 0.43 |
| PPIA | 1 (5) | 0 (0) | 3 (4) | 4 | 1.00 |
| PRKCH | 19 (86) | 14 (93) | 75 (100) | 7 | 0.006 |
| PRKCZ | 10 (46) | 3 (20) | 9 (12) | -8 | 0.003 |
| PTPRN | 0 (0) | 1 (7) | 4 (5) | -2 | 0.63 |
| REG3A | 20 (91) | 15 (100) | 68 (91) | -9 | 0.67 |
| TNFA | 0 (0) | 1 (7) | 4 (5) | -2 | 0.63 |
| TUBA1B | 0 (0) | 1 (7) | 8 (11) | 4 | 0.31 |
| VM | 0 (0) | 3 (20) | 31 (41) | 21 | <0.001 |

AGRIN, agrin; AGT angiotensinogen; ARHGDIB, Rho GDP-dissociation inhibitor 2; AURKA, aurora kinase A-interacting protein; CD36, platelet glycoprotein 4; CXCL9, C-X-C motif chemokine ligand 9; CXCL10, C-X-C motif chemokine ligand 10; CXCL11, C-X-C motif chemokine ligand 11; CHAF1B, chromatin assembly factor 1 subunit B; EIF2A, eukaryotic translation initiation factor 2A; ENO1, alpha-enolase; FLRT2, leucine-rich repeat transmembrane protein 2; GDNF, glial cell line-derived neurotrophic factor; GSTT1, glutathione S-transferase theta 1; GAPDH, glyceraldehyde-3-phosphate dehydrogenase; HNRNPK, heterogeneous nuclear ribonucleoprotein K; IFNG, interferon gamma; IFIH1, interferon-induced helicase C domain-containing protein 1; LMNA, lamin-A/C; LMNB, lamin-B1; MYOSIN, cardiac myosin-binding protein C; NCL, nucleolin; PPIA, peptidyl-prolyl cis-trans isomerase A; PECR, peroxisomal trans-2-enoyl-CoA reductase; PRKCH, protein kinase C eta; PRKCZ, protein kinase C zeta; PTPRN, receptor-type tyrosine-protein phosphatase-like N; REG3A, regenerating islet-derived protein 3-alpha; PLA2R, secretory phospholipase A2 receptor; TNFA, tumor necrosis factor alpha; TUBA1B, tubulin alpha-1B chain; VM, vimentin.

**Table S2. Normalized mean fluorescence intensity (nMFI) of 32 non-HLA antibodies in three independent cohorts: adult healthy controls (HC, n=22), pediatric liver transplant candidates (Pre-Tx, n=15) and long-term liver transplant recipients (Post-Tx, n=75). The change in nMFI (∆nMFI) for each specificity was calculated by subtracting the median nMFI from Pre-Tx to the median nMFI from Post-Tx.**

| **Antibody** | **HC,  median (IQR)** | **Pre-Tx,  median (IQR)** | **Post-Tx,  median (IQR)** | **∆nMFI** | **P-value** |
| --- | --- | --- | --- | --- | --- |
| AGRIN | 0.09 (0.06–0.20) | 0.23 (0.17–0.70) | 0.33 (0.17–0.92) | 0.10 | 0.001 |
| AGT | 0.12 (0.10–0.18) | 0.26 (0.22–0.40) | 0.32 (0.19–0.66) | 0.06 | <0.001 |
| ARHGDIB | 0.11 (0.09–0.16) | 0.20 (0.16–0.47) | 0.27 (0.15–0.46) | 0.07 | <0.001 |
| AURKA | 0.15 (0.12–0.36) | 0.55 (0.32–0.81) | 0.59 (0.29–0.91) | 0.04 | 0.002 |
| CD36 | 0.09 (0.07–0.15) | 0.27 (0.17–0.34) | 0.21 (0.12–0.50) | -0.06 | <0.002 |
| CHAF1B | 0.07 (0.05–0.13) | 0.30 (0.14–0.59) | 0.21 (0.10–0.34) | -0.09 | <0.003 |
| CXCL10 | 0.58 (0.37–1.03) | 1.28 (0.60–3.77) | 1.27 (0.54–2.33) | -0.01 | 0.02 |
| CXCL11 | 0.66 (0.42–0.93) | 2.98 (1.59–3.91) | 1.51 (0.95–2.44) | -1.47 | <0.001 |
| CXCL9 | 0.27 (0.18–0.40) | 1.18 (0.36–1.74) | 0.53 (0.25–0.99) | -0.65 | 0.006 |
| EIF2A | 0.03 (0.02–0.06) | 0.13 (0.04–0.21) | 0.13 (0.05–0.27) | 0.00 | <0.001 |
| ENO1 | 0.05 (0.02–0.15) | 0.11 (0.04–0.24) | 0.17 (0.07–0.38) | 0.06 | 0.005 |
| FLRT2 | 0.18 (0.13–0.22) | 0.50 (0.36–0.74) | 0.45 (0.27–1.12) | -0.05 | <0.001 |
| GADPH | 0.22 (0.16–0.26) | 0.58 (0.52–1.03) | 0.59 (0.30–1.17) | 0.01 | <0.001 |
| GDNF | 0.33 (0.14–0.46) | 0.81 (0.29–1.36) | 0.58 (0.33–1.00) | -0.23 | 0.005 |
| GSTT1 | 0.15 (0.10–0.26) | 0.29 (0.23–0.41) | 0.38 (0.20–0.71) | 0.09 | <0.001 |
| HNRNPK | 1.08 (0.59–2.00) | 2.71 (1.61–4.25) | 2.07 (1.07–4.13) | -0.64 | 0.01 |
| IFIH1 | 0.04 (0.02–0.05) | 0.09 (0.07–0.14) | 0.07 (0.03–0.10) | -0.02 | 0.002 |
| IFNG | 0.29 (0.19–0.64) | 1.22 (0.61–2.69) | 1.54 (0.70–3.11) | 0.32 | <0.001 |
| LMNA | 0.05 (0.03–0.09) | 0.20 (0.15–0.28) | 0.18 (0.12–0.29) | -0.02 | <0.001 |
| LMNB1 | 0.17 (0.13–0.28) | 0.49 (0.32–0.63) | 0.36 (0.20–0.57) | -0.13 | <0.001 |
| MYOSIN | 0.10 (0.07–0.17) | 0.35 (0.20–0.42) | 0.33 (0.23–0.60) | -0.02 | <0.001 |
| NCL | 0.07 (0.06–0.11) | 0.27 (0.11–0.39) | 0.20 (0.11–0.34) | -0.07 | <0.001 |
| PECR | 0.16 (0.05–0.53) | 0.45 (0.24–1.29) | 0.29 (0.12–0.62) | -0.16 | 0.04 |
| PLA2R | 0.07 (0.05–0.12) | 0.16 (0.07–0.20) | 0.10 (0.01–0.20) | -0.06 | 0.17 |
| PPIA | 0.12 (0.04–0.21) | 0.27 (0.11–0.42) | 0.20 (0.11–0.43) | -0.07 | 0.07 |
| PRKCH | 1.60 (1.32–2.24) | 3.57 (3.07–4.19) | 4.47 (3.38–5.50) | 0.90 | <0.001 |
| PRKCZ | 0.63 (0.18–1.37) | 0.33 (0.16–0.49) | 0.27 (0.12–0.68) | -0.06 | 0.08 |
| PTPRN | 0.07 (0.05–0.11) | 0.27 (0.22–0.37) | 0.20 (0.11–0.38) | -0.07 | <0.001 |
| REG3A | 2.77 (1.76–3.42) | 6.37 (2.39–10.78) | 4.24 (2.26–7.83) | -2.13 | 0.01 |
| TNFA | 0.05 (0.04–0.06) | 0.18 (0.07–0.25) | 0.17 (0.07–0.29) | -0.01 | <0.001 |
| TUBA1B | 0.15 (0.10–0.21) | 0.34 (0.28–0.65) | 0.31 (0.18–0.68) | -0.03 | <0.001 |
| VM | 0.16 (0.11–0.31) | 0.40 (0.23–0.79) | 0.65 (0.29–1.90) | 0.25 | <0.001 |

AGRIN, agrin; AGT angiotensinogen; ARHGDIB, Rho GDP-dissociation inhibitor 2; AURKA, aurora kinase A-interacting protein; CD36, platelet glycoprotein 4; CXCL9, C-X-C motif chemokine ligand 9; CXCL10, C-X-C motif chemokine ligand 10; CXCL11, C-X-C motif chemokine ligand 11; CHAF1B, chromatin assembly factor 1 subunit B; EIF2A, eukaryotic translation initiation factor 2A; ENO1, alpha-enolase; FLRT2, leucine-rich repeat transmembrane protein 2; GDNF, glial cell line-derived neurotrophic factor; GSTT1, glutathione S-transferase theta 1; GAPDH, glyceraldehyde-3-phosphate dehydrogenase; HNRNPK, heterogeneous nuclear ribonucleoprotein K; IFNG, interferon gamma; IFIH1, interferon-induced helicase C domain-containing protein 1; LMNA, lamin-A/C; LMNB, lamin-B1; MYOSIN, cardiac myosin-binding protein C; NCL, nucleolin; PPIA, peptidyl-prolyl cis-trans isomerase A; PECR, peroxisomal trans-2-enoyl-CoA reductase; PRKCH, protein kinase C eta; PRKCZ, protein kinase C zeta; PTPRN, receptor-type tyrosine-protein phosphatase-like N; REG3A, regenerating islet-derived protein 3-alpha; PLA2R, secretory phospholipase A2 receptor; TNFA, tumor necrosis factor alpha; TUBA1B, tubulin alpha-1B chain; VM, vimentin.
